# The Association Between Plasma Hyaluronan Level and Plaque Types in ST-Segment–Elevation Myocardial Infarction Patients

**DOI:** 10.3389/fcvm.2021.628529

**Published:** 2021-02-12

**Authors:** Jiannan Li, Yu Tan, Zhaoxue Sheng, Peng Zhou, Chen Liu, Hanjun Zhao, Li Song, Jinying Zhou, Runzhen Chen, Yi Chen, Hongbing Yan

**Affiliations:** ^1^Department of Cardiology, National Center for Cardiovascular Diseases, Fuwai Hospital, Peking Union Medical College, Chinese Academy of Medical Sciences, Beijing, China; ^2^Xiamen Cardiovascular Hospital, Xiamen University, Fujian, China; ^3^Fuwai Hospital, Chinese Academy of Medical Sciences, Shenzhen, China

**Keywords:** hyaluronan, optical coherence tomography, plaque erosion, ST segment elevated myocardial infarction, CD44

## Abstract

**Background:** The metabolism of hyaluronan (HA) is widely known to be involved in the process of acute coronary syndrome, but it is unknown how circulating HA levels change in ST-Segment–Elevation Myocardial Infarction (STEMI) patients and whether HA is associated with plaque morphology, including rupture and erosion.

**Objectives:** This study focused on the changes in the plasma levels of high molecular weight (HMW) HA (>35 kDa) and CD44 in STEMI patients and their relationship with plaque morphology evaluated by optical coherence tomography (OCT).

**Methods:** We prospectively enrolled 3 cohorts in this study, including 162 patients with STEMI, 34 patients with stable coronary artery disease (S-CAD) and 50 healthy controls. Plaque morphology was detected by OCT analysis, and the plasma levels of HMW HA and CD44 were examined by enzyme-linked immunosorbent assay (ELISA). We compared plasma level of HMW HA and CD44 among STEMI patients, S-CAD patients and healthy controls, as well as in plaque rupture and plaque erosion.

**Results:** The plasma levels of HMW HA and CD44 were significantly lower in STEMI patients than in healthy controls (*p* = 0.009 and *p* < 0.001, respectively). In addition, plasma level of HMW HA in plaque erosion was significantly lower than that in plaque rupture (*p* = 0.021), whereas no differences were found in plasma level of soluble CD44 between plaque rupture and erosion.

**Conclusions:** Low levels of circulating HMW HA and CD44 were independently correlated with STEMI, and low levels of HMW HA were associated with plaque erosion compared with rupture. Moreover, plasma HMW HA might be a useful biomarker for identifying plaque erosion to improve the risk stratification and management of STEMI patients.

## Introduction

An increasing number of studies have demonstrated that plaque rupture is not the only cause of ST-Segment–Elevation Myocardial Infarction (STEMI) (1). Nearly one-third of patients with STEMI have plaque erosion in their culprit lesion (2), which is characterized by a higher concentration of hyaluronan (HA) and versican, with considerably less decorin and biglycan, which exhibit morphological characteristics associated with stability (3). In addition, CD44, a cell surface receptor of HA, prominently localizes to eroded plaques more than in ruptured plaques, pointing again to a distinct mechanistic pathway for erosion (3, 4). However, the mechanism of plaque erosion has not been totally elucidated.

HA is a ubiquitous non-sulfated glycosaminoglycan that exists in nearly all tissues. HA plays an important role in many physiological processes, and it can be activated during pathological conditions such as inflammation and cancer (5). It has also been proven that HA modulates the progression of atherosclerotic plaques in cardiovascular disease, including macrophage retention and matrix proliferation (6). Recent animal experiments showed that plaque erosion was triggered by disturbed blood flow, which was involved in HA metabolism (7). Furthermore, an *in vivo* experiment found that hyaluronidase 2 (HYAL2) and its receptor mediated the recruitment of polymorphonuclear (PMNc) leukocytes and the formation of thrombi in plaque erosion was induced by flow perturbation (8). However, whether systemic HA changes in STEMI is unknown. Moreover, the difference in circulating HA level between plaque rupture and erosion has not yet been investigated.

Optical coherence tomography (OCT), with an extremely high resolution near infrared light-based intravascular imaging modality, enables an accurate identification of plaque morphology *in vivo* (9). This study not only explores how circulating HA changes in STEMI but also provides potential biomarkers and a clinical risk stratification for plaque erosion determined by OCT.

## Methods

### Study Population and Design

**Figure 1:**
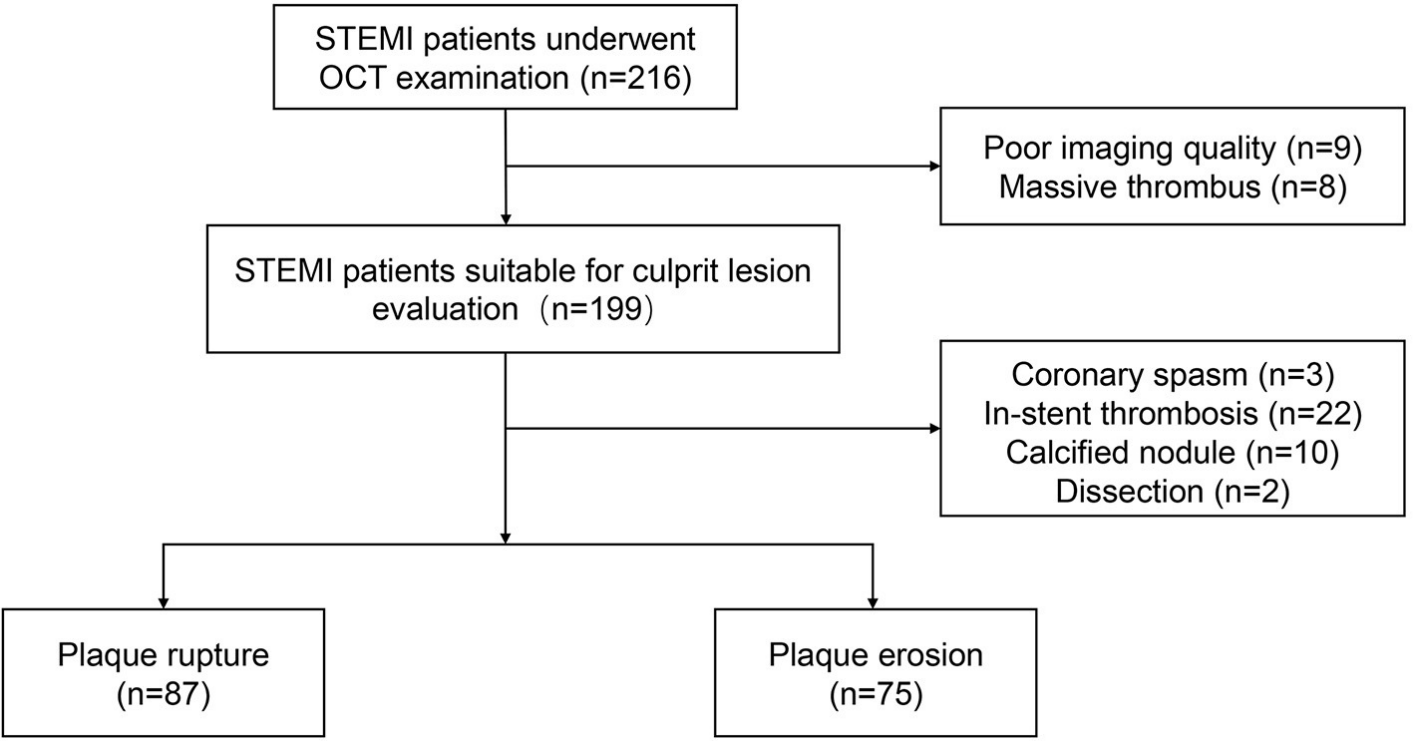
Study flow chart. OCT, optical coherence tomography; STEMI, ST-Segment–Elevation Myocardial Infarction.

We prospectively enrolled 3 cohorts for this study. The first cohort comprised sequential patients (age ≥18 years) who presented with STEMI and underwent emergency procedures at Fuwai Hospital. The culprit lesions of these patients were evaluated using OCT before the interventional procedures. STEMI was defined as continuous chest pain lasting >30 min, ST-Segment–Elevation >0.1 mV in at least 2 contiguous leads or new left bundle-branch block on the 18-lead electrocardiogram (ECG), and an elevated troponin I level (10). Patients with cardiac shock, congestive heart failure, a history of coronary artery bypass graft, liver disease or malignant tumor were excluded. Additionally, those with left main diseases, extremely tortuous or heavily calcified vessels, or chronic total occlusion were excluded owing to the difficulty in performing OCT. Between May 2017 and September 2018, a series of 216 eligible patients with STEMI underwent OCT and were enrolled in our study cohort. The study flow chart is displayed in Figure 1. The second cohort examined was an independent set of 50 prospectively recruited individuals (age ≥18 years) without known cardiovascular diseases from health screens to provide normal HA and CD44 reference interval values. The third cohort includes 34 patients diagnosed as S-CAD according their symptoms and coronary angiographical findings (baseline characteristics in Supplementary Table 1 in the Data Supplement). This study was performed in accordance with the Declaration of Helsinki and was approved by the Ethics Committee of Fuwai Hospital. All patients provided written informed consent.

### Acquisition of OCT Images

Patients were administered 300 mg of aspirin, 180 mg of ticagrelor, or 600 mg of clopidogrel, and 100 IU/kg of heparin before the interventional procedure. Percutaneous coronary intervention was performed *via* radial or femoral access. Thrombus aspiration was used to reduce the thrombus burden and restore antegrade coronary flow. OCT images of the culprit lesions were acquired with the frequency domain ILUMIEN OPTIS OCT system and a dragon fly catheter (St. Jude Medical, Westford, MA) after antegrade blood flow was restored, according to the intracoronary imaging technique described previously.

### OCT Image Analysis

**Figure 2:**
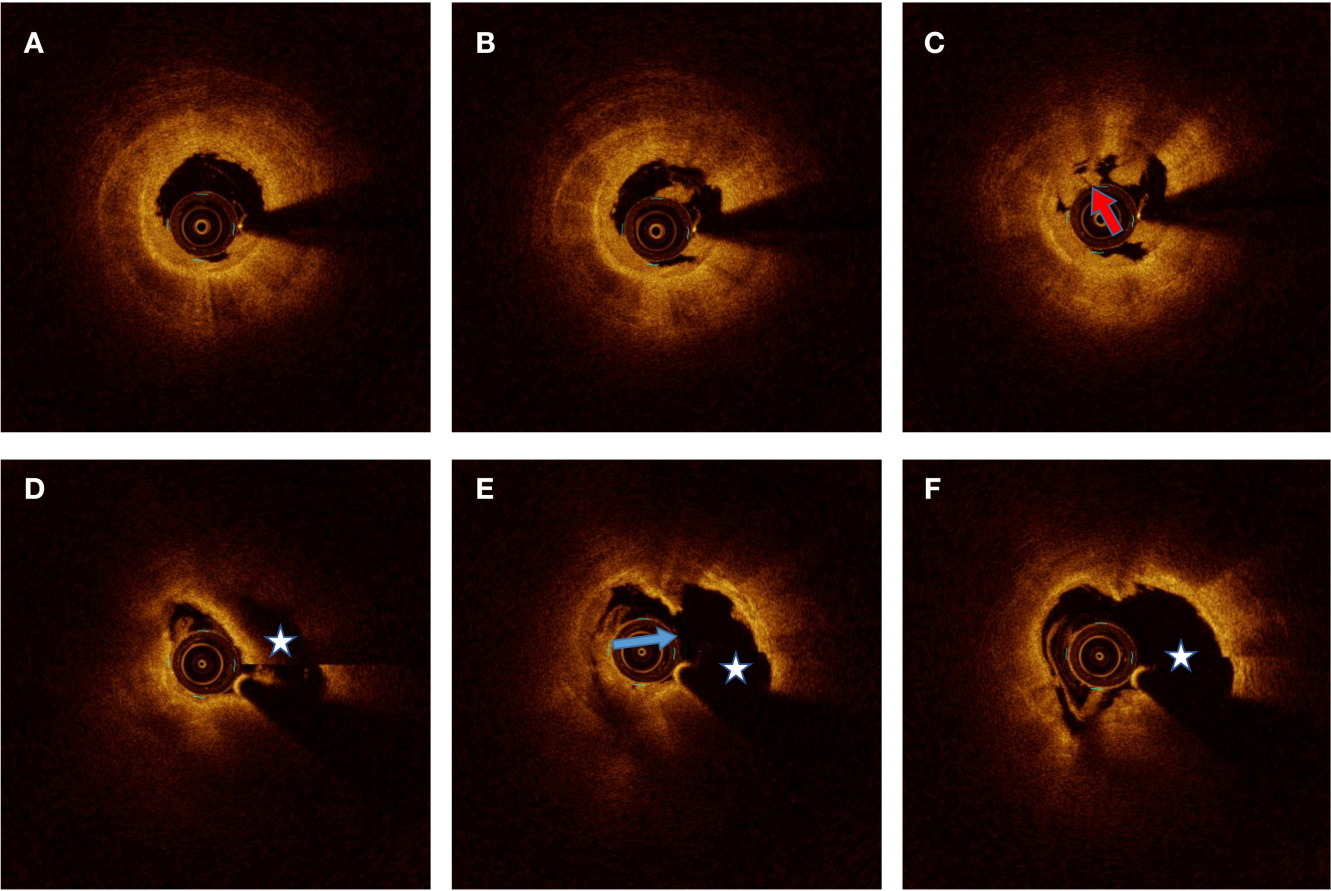
Representative optical coherence tomography images for plaque erosion and rupture. **(A–C)** Plaque erosion defined by residual white thrombus (red arrow) underlying a fibrous plaque without evidence of fibrous cap disruption. **(D–F)** Plaque rupture identified by the presence of disrupted fibrous cap (blue arrow) and cavity formation (pentagram).

All OCT images were anonymously analyzed on a St. Jude OCT Offline Review Workstation by 3 independent investigators who were blinded to the other data (11). The first investigator was primarily responsible for screening suitability for culprit-plaque evaluation. The other two investigators analyzed OCT images. The intra-observer kappa coefficients for plaque rupture and plaque erosion were 0.963 and 0.938, respectively. The inter-observer kappa coefficients for plaque rupture and plaque erosion was 0.950. Inconsistent results were resolved by consensus with investigators who were blinded to the HA and CD44 results. According to previously established criteria (12), plaque rupture was identified by a disrupted fibrous cap with a clear cavity formation (Figure 2). Plaque erosion was identified by the presence of an attached thrombus overlying an intact and visible plaque, luminal surface irregularity of the plaque in the absence of a thrombus, or attenuation of the underlying plaque by the thrombus without superficial lipid or calcification proximal or distal to the thrombus (Figure 2).

### Laboratory Tests

Blood samples were collected *via* radial or femoral access before heparinization using vacutainer tubes containing EDTA. Samples were maintained at 4°C, processed within 3 h, and then stored at −80°C until further analysis. Plasma levels of HA and CD44 were determined by enzyme-linked immunosorbent assay (ELISA) using a Quantikine Hyaluronan Immunoassay kit (DHYAL0) (R&D Systems, Abingdon, UK) which can only test the HA>35 kDa and Human CD44 Elisa Kit (Abcam, Cambridge UK), in accordance with the protocol supplied by the manufacturer (13).

### Statistical Analysis

Continuous data are presented as the mean ± SD or median (interquartile range). Student's *t*-test or a non-parametric test was used for statistical comparisons. One-way analysis of variance (ANOVA) was used to assess differences of continuous data of clinical parameters among the three or more groups. Categorical variables are presented as the count (percent); comparisons between groups were made with the χ^2^-test or Fisher's exact test. Logistic regression analysis was performed to determine the odds ratio (OR) and 95% confidence interval (CI) for plaque erosion stratified according to HA as a categorical variable. Adjustments were made for traditional risk factors (including age, sex, hypertension, diabetes mellitus, smoking, and low-density lipoprotein-cholesterol (LDL-C), high-density lipoprotein-cholesterol (HDL-C), triglyceride level), high sensitive C-reactive protein (hs-CRP) level, estimated glomerular filtration rate (eGFR), and body mass index (BMI). The area under the receiver operating characteristic (ROC) curves (AUC), sensitivity, and specificity were calculated to evaluate the predictive ability of HA for plaque erosion. A 2-tailed *P* < 0.05 was considered statistically significant. The statistical analyses were performed using SPSS software, version 25 (IBM, Armonk, NY).

## Result

### Patient Characteristics

Of the 216 patients with STEMI who underwent OCT examination, 17 patients were excluded because of massive thrombus (*n* = 8) or poor imaging quality (*n* = 9). The remaining 199 patients were suitable for plaque morphology evaluation; 87 patients had plaque rupture, and 75 had plaque erosion. The baseline patient characteristics are displayed in Table 1 for the entire study cohort and categorized by plaque morphology. The mean age of the cohort was 57.3 years, 85.2% were men, 57.4% had hypertension, and 29.6% had diabetes mellitus. Patients with plaque erosion were more likely to be younger and have better renal function than patients with plaque rupture. There were no significant differences in the other clinical variables between the plaque rupture and plaque erosion groups. Then Baseline characteristics were compared among three groups of STEMI, S-CAD and healthy controls. The incidence of nearly all the traditional risk factors including hypertension, diabetes, hyperlipidemia and smoker was significantly higher in STEMI and S-CAD than healthy controls (Table 2).

**Table 1:** Baseline characteristics of subjects stratified by plaque types.

**Variables**	**Total (*n* = 162)**	**Plaque type**		***p*-value**
		**Rupture (*n* = 87)**	**Erosion (*n* = 75)**	
Age (years)	57.3 ± 11.5	59.3 ± 12.1	55.1 ± 10.2	0.021
Males	138 (85.2%)	76 (87.4%)	62 (82.7%)	0.402
Body mass index (kg/m^2^)	26.5 ± 4.0	26.4 ± 4.5	26.5 ± 3.4	0.948
Diabetes mellitus	48 (29.6%)	27 (31%)	21 (28%)	0.673
Hypertension	93 (57.4%)	54 (62.1%)	39 (52%)	0.196
Hyperlipidemia	132 (81.5%)	75 (86.2%)	57 (76%)	0.095
Ischemic stroke	12 (7.4%)	8 (9.2%)	4 (5.3%)	0.349
Smoker	113 (69.8%)	61 (70.1%)	52 (69.3%)	0.914
Hs-CRP (mg/L)	6.3 (2.6–11.0)	7.2 (2.6–11.1)	5.3 (2.6–10.9)	0.593
eGFR (ml/min/1.732 m^2^)	88.4 ± 21.2	83.3 ± 20.8	94.3 ± 20.2	0.001
Glycosylated hemoglobin	6.7 ± 1.7	6.6 ± 1.6	6.8 ± 1.8	0.538
Triglyceride (mmol/L)	1.4 (0.8–2.0)	1.3 (0.8–1.9)	1.5 (0.9–2.1)	0.273
LDL-C (mmol/L)	2.7 ± 0.9	2.6 ± 0.9	2.9 ± 0.8	0.066
HDL-C (mmol/L)	1.1 (0.9–1.2)	1.1 (0.9–1.2)	1.1 (1.0–1.3)	0.149
Plasma hyaluronan (ng/ml)	30.1 (17.6–47.7)	36.2 (18.9–51.9)	25.1 (15.4–41.4)	0.021
Plasma CD44 (ng/ml)	143.7 (126.2–162.8)	142.5 (129.1–162.6)	144.5 (123.4–164.7)	0.975

*Continuous data are presented as mean ± SD or median (interquartile range), categorical variables are presented as %. eGFR, estimated glomerular filtration rate; HDL-C, high-density lipoprotein-cholesterol; LDL-C, low-density lipoprotein-cholesterol; hs-CRP, high sensitive C-reactive protein*.

**Table 2:** Baseline characteristics of subjects among STMEI, S-CAD and healthy controls.

**Variables**	**STEMI (*n* = 162)**	**S-CAD (*n* = 34)**	**HCs (*n* = 50)**	***p*-value**
Age (years)	57.3 ± 11.5	61.9 ± 8.4	57.9 ± 9.3	0.076
Males	138 (85.2%)	21 (61.8%)	31 (62.0%)	<0.001
BMI (kg/m^2^)	26.5 ± 4.0	25.0 ± 3.6	24.4 ± 2.9	0.001
Diabetes mellitus	48 (29.6%)	16 (47.1%)	3 (6.0%)	<0.001
Hypertension	93 (57.4%)	23 (67.6%)	11 (22.0%)	<0.001
Hyperlipidemia	132 (81.5%)	30 (82.2%)	25 (50.0%)	<0.001
Ischemic stroke	12 (7.4%)	7 (20.6%)	1 (2.0%)	<0.001
Smoker	113 (69.8%)	18 (52.9%)	6 (12.0%)	<0.001
Hs-CRP (mg/L)	6.3 (2.6–11.0)	1.1 (0.6–2.6)	0.7 (0.4–1.3)	<0.001
eGFR (ml/min/1.732 m^2^)	88.4 ± 21.2	80.5 ± 17.4	92.9 ± 19.3	0.025
Triglyceride (mmol/L)	1.4 (0.8–2.0)	1.3 (0.9–1.8)	1.2 (0.8–1.6)	0.058
LDL-C (mmol/L)	2.7 ± 0.9	2.2 ± 1.0	2.9 ± 0.9	0.001
HDL-C (mmol/L)	1.1 (0.9–1.2)	1.2 (1.0–1.5)	1.5 (1.3–1.7)	<0.001
Plasma HA (ng/ml)	30.1 (17.6–47.7)	36.5 (18.3–67.8)	39.3 (26.0–53.7)	0.004
Plasma CD44 (ng/ml)	143.7 (126.2–162.8)	178.7 (160.1–191.3)	172.1 (156.4–197.4)	<0.001

*Continuous data are presented as mean ± SD or median (interquartile range), categorical variables are presented as %. STEMI, ST-segment–elevation myocardial Infarction; S-CAD, stable coronary artery disease; HCs, healthy controls; BMI, body mass index; hs-CRP, high sensitivity C reactive protein; LDL-C, low density lipoprotein cholesterol; HDL-C, high density lipoprotein cholesterol; eGFR, estimated glomerular filtration rate; HA, hyaluronan*.

**Figure 3:**
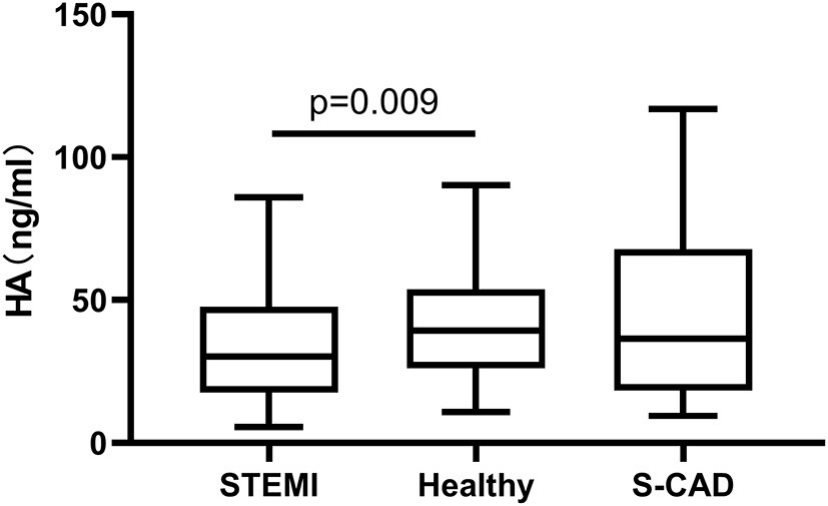
Relations of plasma HA levels among patients with STEMI, S-CAD and healthy subjects. HA, hyaluronan; STEMI, ST-Segment–Elevation Myocardial Infarction; S-CAD, Stable Coronary Artery Disease.

**Figure 4:**
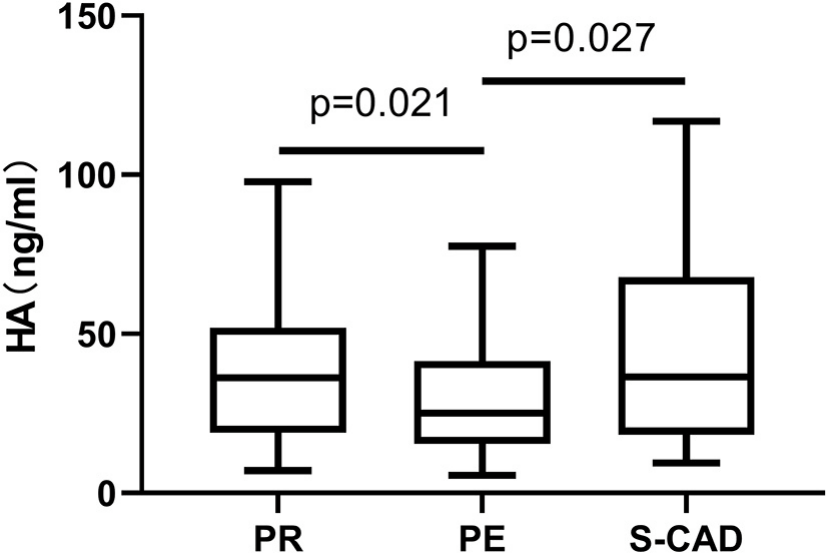
Relations of plasma HA levels among patients with PR, PE, and S-CAD. HA, hyaluronan; PR, Plaque Rupture; PE, Plaque Erosion; S-CAD, Stable Coronary Artery Disease.

**Figure 5:**
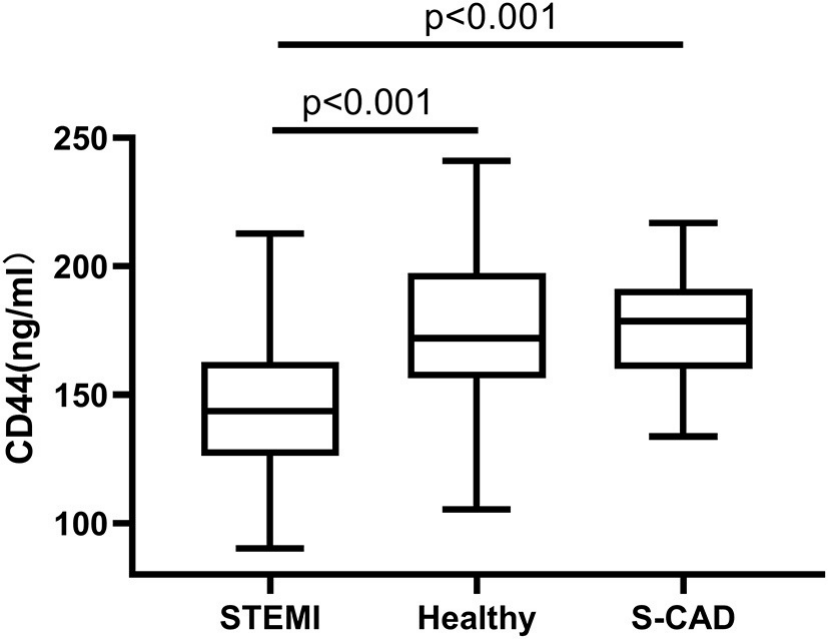
Relations of plasma CD44 levels among patients with STEMI, S-CAD and healthy subjects. HA, hyaluronan; STEMI, ST-Segment–Elevation Myocardial Infarction; S-CAD, Stable Coronary Artery Disease.

We first performed a cross-sectional comparison of the HA levels among the whole STEMI cohort, an independent set of 50 prospectively recruited individuals without known cardiovascular disease and 34 patients diagnosed as S-CAD. We observed that plasma HA levels (>35 kDa) were significantly lower in the patients with STEMI than in the healthy controls [30.1 ng/ml (17.6–47.7) vs. 39.3 ng/ml (26.0–53.7) *p* = 0.009], but there is no significant difference of HA levels between patients with STEMI and S-CAD (Figure 3). When we distributed patients with STEMI into plaque rupture (PR) and plaque erosion (PE), we found HA levels of PE is significantly lower than PR and S-CAD [25.1 ng/ml (15.4–41.4) vs. 36.2 ng/ml (18.9–51.9) *p* = 0.021; 25.1 ng/ml (15.4–41.4) vs. 36.5 ng/ml (18.3–67.8) *p* = 0.027, respectively] (Figure 4). Then we compared plasma CD44 concentration among these three groups. We found that CD44 levels in patients with STEMI is significantly lower than healthy controls and patients with S-CAD [143.7 ng/ml (126.2–162.8) vs. 172.1 ng/ml (156.4–197.4) *p* < 0.001; 143.7 ng/ml (126.2–162.8) vs. 178.7 ng/ml (160.1–191.3) *p* < 0.001, respectively] (Figure 5). However, subgroup analysis showed that plasma CD44 levels are not significantly different between patients with PR and those with PE [142.5 ng/ml (129.1–162.6) vs. 144.5 ng/ml (123.4–164.7), *p* = 0.975] (Table 1).

**Figure 6:**
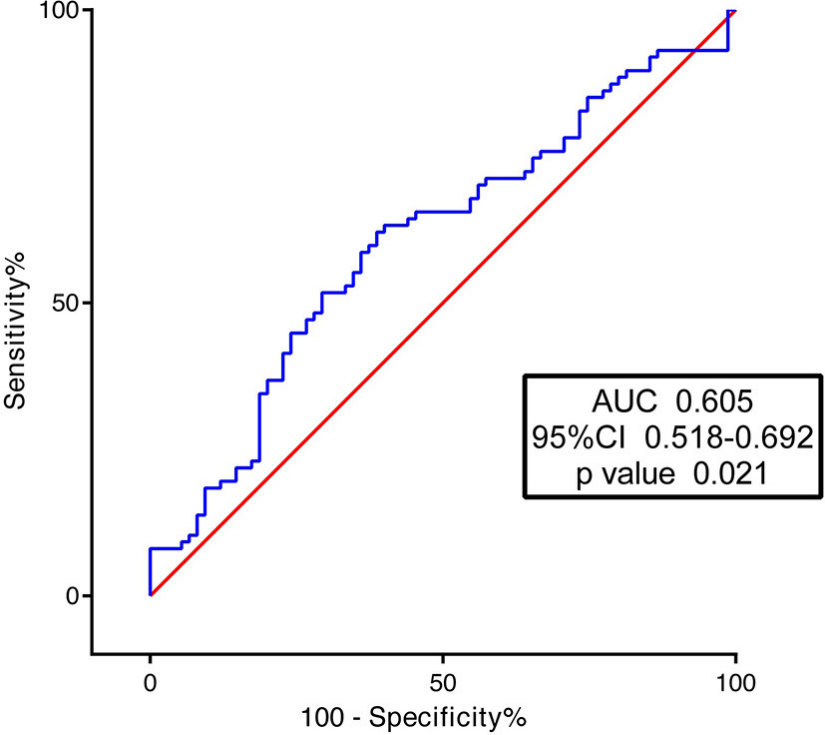
Receiver operating characteristic curves of hyaluronan (HA) for predicting plaque erosion.

According to the ROC analysis, the AUC of HA levels in discriminating plaque erosion from rupture was 0.605 (95% CI 0.518–0.692, *p* = 0.021) (Figure 6). The optimal cutoff value was 29.6 ng/ml. In logistic regression analysis, HA was transformed to categorical variables through cutoff value (29.6 ng/ml). After adjustment of age, sex, BMI, history of diabetes mellitus, hypertension, hyperlipidemia, smoking, LDL-C, HDL-C, triglyceride, eGFR, hs-CRP, and CD44, HA levels were independently associated with plaque types (Table 3).

**Table 3:** Logistic regression analyses of plasma HA levels in patients with plaque rupture and erosion.

**Variables**	**Univariate**		**Multivariate**	
	**OR (95%CI)**	***p*-value**	**OR (95%CI)**	***p*-value**
Age (years)	1.033 (1.005–1.063)	0.023	0.981 (0.945–1.018)	0.318
Sex (male)	0.690 (0.289–1.648)	0.404	1.207 (0.395–3.694)	0.741
Body mass index (kg/m^2^)	0.997 (0.924–1.077)	0.947	1.040 (0.945–1.145)	0.418
Smoking	1.038 (0.530–2.032)	0.914	1.054 (0.442–2.514)	0.906
Hypertension	1.510 (0.807–2.827)	0.197	0.971 (0.449–2.097)	0.940
Diabetes	1.157 (0.587–2.281)	0.673	1.026 (0.466–2.259)	0.948
Hyperlipidemia	0.507 (0.226–1.136)	0.099	0.393 (0.150–1.029)	0.057
LDL-C (mmol/L)	0.709 (0.489–1.026)	0.068	1.050 (0.677–1.630)	0.827
HDL-C (mmol/L)	3.694 (1.027–13.286)	0.045	5.436 (1.008–29.306)	0.049
Triglyceride (mmol/L)	1.004 (0.804–1.253)	0.975	0.964 (0.732–1.271)	0.796
Hs-CRP (mg/L)	1.025 (0.957–1.097)	0.479	1.000 (0.924–1.082)	1.000
eGFR	0.974 (0.958–0.990)	0.001	1.024 (1.004–1.044)	0.017
HA (ng/ml)	2.596 (1.375–4.899)	0.003	2.616 (1.246–5.496)	0.011
CD44 (ng/ml)	1.003 (0.995–1.012)	0.464	1.011 (0.999–1.024)	0.073

*LDL-C, low-density lipoprotein-cholesterol; HDL-C, high-density lipoprotein-cholesterol; hs-CRP, high sensitive C-reactive protein; eGFR, estimated glomerular filtration rate; HA, hyaluronan; HA was transformed to categorical variables through cutoff value (29.6 ng/ml)*.

## Discussion

Currently, with the development of new technologies for imaging and pathology, we have further insight into the mechanism of myocardial infarction. Myocardial infarction can be divided into different plaque types according to OCT examination, and it is important to distinguish plaque rupture from erosion because patients with different plaque types have different risks and prognoses (14). Patients with erosion may benefit from pharmacological therapy rather than mechanical revascularization (15). It is more convenient to determine the types of plaque by biomarkers rather than OCT, which is more expensive for patients. Our previous study revealed that plasma trimethylamine N-oxide (TMAO) can be a useful biomarker to predict plaque rupture (16). However, although the mechanism of plaque erosion has been partly studied, there is still no acknowledged biomarker for predicting plaque erosion. In this study, we first reported the distinction of plasma HA and CD44 between patients with STEMI and healthy people. Additionally, we found that plasma HA levels in erosion patients were significantly lower than in rupture, S-CAD patients and healthy subjects, which meant that plasma HA levels can be used as a potential biomarker to predict plaque erosion.

The incidence of PE might not be influenced by conventional risk factors which is quite distinct from PR. A clinical study showed that the presence of plaque erosion was related to anterior ischemia, no diabetes and normal renal function (17). The early study enrolled 209 patients with PE also demonstrated that ACS patients with less dyslipidemia, hypertension, CKD or diabetes tended to be PE (18). In our study, younger patients with better renal function were susceptible to PE. Although histories of hypertension and dyslipidemia had no significant difference between PR and PE, the prevalence of them was lower in patients with PE in our study cohort, which showed the same trend with previous study. Plenty of Imaging and experimental study revealed that pathobiology of PE was mainly involved with endothelial activation and fluid dynamics factors (17). A recent research based on OCT showed that high shear stress might play an important role in initiating thrombotic processes of PE (19). Furthermore, a growing number of studies have revealed that HA and its correlated enzyme also took part in the mechanism of PE mediated by fluid dynamic change (8).

HA plays a crucial role in the progression of cardiovascular disease and is involved in several important phases of coronary artery disease (CAD), such as inflammation and angiogenesis (20). HA exists in plasma in two forms: high molecular weight (HMW)-HA and low molecular weight (LMW)-HA. At homeostasis, HA is predominantly in its high molecular mass form of over 1,000 kDa. However, in tissue injury or inflammation, HYAL2 and HYAL1 are upregulated to break down HA to 20 kDa, which binds to the receptors of immune cells (21). It has been reported that HYAL2-deficient mice display a significant increase in plasma HA levels (22). HYAL2 and CD44 exist in many kinds of components in blood, such as platelets and monocytes, all of which contribute to HMW-HA degradation when inflammation occurs. Furthermore, the metabolism of HA is influenced by not only inflammation but also alteration of blood flow, which is related to plaque erosion according to the current study (23). A recent study demonstrated that plaque erosion was associated with high endothelial shear stress (ESS), spatial ESS gradient (ESSG), and oscillatory shear index (OSI). Particularly, the OSI in PE was significantly higher than PR (19, 24). Meanwhile, an experimental study suggested that hyaluronan expression on the endothelial surface was reduced when exposed to oscillatory flow, which was regulated by Krüppel-like Factor 2 (25). It seemed that high OSI was the initial factor of plaque erosion as well as alteration of HA metabolism. Additionally, an increasing number of clinical investigations have found that HA was correlated with the pathogenesis of plaque erosion, and pathological observations have shown that eroded plaques have few inflammatory cells but abundant proteoglycan and glycosaminoglycans, including HA and its receptor CD44 (26). A recent clinical study revealed that the expression of HYAL2 and CD44 in peripheral blood mononuclear cells (PBMCs) increased in acute coronary syndrome (ACS), especially in patients with plaque erosion compared with stable CAD and healthy people (8).

However, most previous studies focused on HA inside the plaque, not in circulation. The source of circulating HA may come from many kinds of cells including endothelial cell, stroma cell and atherosclerotic plaque (6). Interestingly, our study found that plasma HA levels decreased in STEMI patients, particularly in plaque erosion. Although concentration of circulating HA might be influenced by many factors including diabetes, smoking and inflammatory conditions (27, 28), after adjustment of traditional risk factors, the plasma level of HA was still lower in PE than PR. This may indicate that alteration of regional endothelial shear stress and eroded plaques independently influenced the systemic change in HA levels in STEMI patients. Nevertheless, another study found that the plasma level of HA was significant higher in acute myocardial infarction group than normal, which was on contrary to our result (29). It is worth noting that we only examined the HA of which molecular size was larger than 35 kDa due to the limitation of ELISA kit. Due to the biological properties of HA, this difference can be explained by degradation of HWM-HA into LWM-HA in ischemia and inflammation condition (5). Actually, thanks to the various boundary and sensitivity of different HA ELISA kit, the value of HA also differed largely among different studies (13, 27, 30). Thus, it needs further investigation that how the plasma level of HA changed in PE which is lower than 35 kDa.

According to the basic research, reduction of plasma level of HA(>35 kDa) in plaque erosion might be involved with many pathophysiological processes including Toll-like receptor 2 (TLR2) stimulation, endothelial activation and neutrophil accumulation. Previous studies found that TLR2 is widely expressed on the surface of eroded plaques in the zone of flow perturbation (31). As one of the endogenous ligands, HA activate TLR2 which contributes to endothelial cell detachment and apoptosis (32). Meanwhile, HA can bind to immune cells, such as macrophages and neutrophils, and then eventually being degraded *via* the HYAL2 and CD44 pathways. In plaque erosion, overexpression of CD44 induces the adhesion of neutrophils to HA (7). HA fragmentation can result from degradation by the reactive oxygen species (ROS) that are produced by neutrophils (33). On the other hand, there is evidence that macrophages are involved in HA uptake and the removal of HA fragments in inflammation (34). Additionally, platelet aggregation may also participate in degradation of HA in STEMI patients because HYAL2 becomes expressed on the cell surface of activated platelets and have high hyaluronidase activity (35). Moreover, platelets also express CD44, which can bind to free HA in plasma when it is activated (36).

Furthermore, in our study, the plasma level of CD44 was also decreased in STEMI patients compared with healthy controls. In reference with a previous study, it was speculated that plasma HA might bind to the N-terminal hyaluronan binding domain (HABD) of CD44 in an inflammatory state to consume free CD44 in plasma (37). Although the mRNA expression of CD44v1 and CD44v6 in PBMCs has been reported to be different between plaque rupture and erosion in the past (8), there was no difference in soluble CD44 between plaque erosion and rupture in our study. Soluble CD44 is not only regulated by HA but also other factors, such as cytokines and shedding from immune cells (38). The metabolism of soluble CD44 in STEMI patients needs further investigation.

It has been reported that the inhibition of HA synthesis accelerates the process of atherosclerosis because HA provides a protective effect on blood vessels (39). Therefore, the relationship between HA reduction and atherosclerosis may exist as a circle of positive feedback. However, the continuous production of HA has a compensatory function. HA is produced by stromal cells *via* hyaluronan synthases (HAS1-3) (5). A recently published study found that the gene expression of CD44 and HYAL2 are different between plaque rupture and erosion, but they can return to baseline after 1 year of follow-up (8). This finding implies that HA has a compensatory effect but that this effect does not occur rapidly. In the acute phase of ischemia and inflammation, the level of HA decreased, but whether it can recover to normal needs further investigation.

This study has several potential limitations. First, patients with cardiac shock, congestive heart failure, a history of coronary artery bypass graft, left main diseases, extremely tortuous or heavily calcified vessels, or chronic total occlusion were not enrolled in our study. In addition, patients with massive thrombi have poor image quality. Therefore, selection bias cannot be excluded. Second, there is no independent cohort to validate the predictive value of HA in discriminating between plaque morphologies, which we aim to include in future studies. Third, hyaluronan ELISA kit (DHYAL0, R&D Systems, Abingdon, UK) is only able to test the hyaluronan>35 kDa, and the circulating level of LMW-HA which is lower than 35 kDa is unknown. In the future, we hope to use mass spectrometer to separate different molecular weight of HA. Forth, we only observed the plaque rupture and plaque erosion in STEMI patients but did not analyze thin-cap fibroatheroma. Fifth, the source of circulating HA in our study remains unclear. Finally, the AUC of plasma HA levels to predict erosion is low, which may be related to the small sample size. We hope to expand the number of enrolled patients in our further investigation or explore other biomarkers which combine with HA to elevate the AUC.

## Conclusion

To the best of our knowledge, this study is the first to demonstrate an independent association of low plasma HA levels with plaque erosion using OCT in patients with STEMI, which may become a potential biomarker or provide novel therapeutic targets for plaque erosion.

## Data Availability Statement

The data used to support the findings of this study are available from the corresponding author upon request.

## Ethics Statement

The studies involving human participants were reviewed by and complied with the principles of the Declaration of Helsinki, and was approved by the Review Board at Fuwai Hospital. The patients/participants provided their written informed consent to participate in this study.

## Author Contributions

JL and YT analyzed and interpreted the complete data and were major contributors in writing the manuscript. ZS contributed to the analysis of baseline HA concentration. PZ and CL played a leading role in patient enrolment and conducting the registry study. JZ, YC, and RC collected and analyzed the patient data regarding clinical characteristics. HY, LS, and HZ supervised the study and were responsible in funding support. All authors read and approved the final manuscript.

## Conflict of Interest

The authors declare that the research was conducted in the absence of any commercial or financial relationships that could be construed as a potential conflict of interest.
