# Supplementary material for: The Association Between Plasma Hyaluronan Level and Plaque Types in ST-Segment–Elevation Myocardial Infarction Patients
Source: Front Cardiovasc Med. 2021 Feb 12;8:628529. doi: 10.3389/fcvm.2021.628529 (PMC7907588; doi:10.3389/fcvm.2021.628529)
Supplement: Supplementary file 1 [file Table_1.docx]

Supplementary table 1. Baseline characteristics of healthy controls, S-CAD, STEMI PE and STEMI PR patients.

| Variables | Rupture (n=87) | Erosion (n=75) | S-CAD（n=34） | HCs（n=50） | p-value |
| --- | --- | --- | --- | --- | --- |
| Age (years) | 59.3±12.1 | 55.1±10.2 | 61.9±8.4 | 57.9±9.3 | 0.149 |
| Males | 76(87.4%) | 62(82.7%) | 21(61.8%) | 31(62.0%) | 0.001 |
| BMI (kg/m2) | 26.4±4.5 | 26.5±3.4 | 25.0±3.6 | 24.4±2.9 | 0.003 |
| Diabetes mellitus | 27(31%) | 21(28%) | 16(47.1%) | 3(6.0%) | <0.001 |
| Hypertension | 54(62.1%) | 39(52%) | 23(67.6%) | 11(22.0%) | <0.001 |
| Hyperlipidemia | 75(86.2%) | 57(76%) | 30(82.2%) | 25(50.0%) | <0.001 |
| Ischemic stroke | 8(9.2%) | 4(5.3%) | 7(20.6%) | 1(2.0%) | 0.020 |
| Smoker | 61(70.1%) | 52(69.3%) | 18(52.9%) | 6(12.0%) | <0.001 |
| Hs-CRP (mg/L) | 7.2(2.6-11.1) | 5.3(2.6-10.9) | 1.1(0.6-2.6) | 0.7(0.4-1.3) | <0.001 |
| eGFR (ml/min/1.732m^2^) | 83.3±20.8 | 94.3±20.2 | 80.5±17.4 | 92.9±19.3 | 0.028 |
| Triglyceride (mmol/L) | 1.3(0.8-1.9) | 1.5(0.9-2.1) | 1.3(0.9-1.8) | 1.2(0.8-1.6) | 0.136 |
| LDL-C (mmol/L) | 2.6±0.9 | 2.9±0.8 | 2.2±1.0 | 2.9±0.9 | 0.001 |
| HDL-C (mmol/L) | 1.1(0.9-1.2) | 1.1(1.0-1.3) | 1.2(1.0-1.5) | 1.5(1.3-1.7) | <0.001 |
| Plasma HA (ng/ml) | 36.2(18.9-51.9) | 25.1(15.4-41.4) | 36.5(18.3-67.8) | 39.3(26.0-53.7) | 0.004 |
| Plasma CD44 (ng/ml) | 142.5(129.1-162.6) | 144.5(123.4-164.7) | 178.7(160.1-191.3) | 172.1(156.4-197.4) | <0.001 |

Continuous data are presented as mean ± SD or median (interquartile range), categorical variables are presented as %. S-CAD, stable coronary artery disease; HCs, healthy controls; BMI, body mass index; hs-CRP, high sensitivity C reactive protein; LDL-C, low density lipoprotein cholesterol; HDL-C, high density lipoprotein cholesterol; eGFR, estimated glomerular filtration rate; HA, hyaluronan.
